# Congenital microcephaly unrelated to flavivirus exposure in coastal Kenya

**DOI:** 10.12688/wellcomeopenres.15568.1

**Published:** 2019-11-15

**Authors:** Hellen C. Barsosio, John N. Gitonga, Henry K. Karanja, Doris K. Nyamwaya, Donwilliams O. Omuoyo, Everlyn Kamau, Mainga M. Hamaluba, Joyce U. Nyiro, Barnes S. Kitsao, Amek Nyaguara, Stella Mwakio, Charles R. Newton, Rosemary Sang, Daniel Wright, Eduard J. Sanders, Anna C. Seale, Charles N. Agoti, James A. Berkley, Philip Bejon, George M. Warimwe

**Affiliations:** 1 KEMRI-Wellcome Trust Research Programme, Kilifi, Kenya; 2 Liverpool School of Tropical Medicine, Liverpool, UK; 3 Department of Psychiatry, University of Oxford, Oxford, UK; 4 KEMRI-Centre for Virus Research, Nairobi, Kenya; 5 The Jenner Institute, University of Oxford, Oxford, UK; 6 London School of Hygiene & Tropical Medicine, London, UK; 7 Centre for Tropical Medicine & Global Health, University of Oxford, Oxford, UK

**Keywords:** Congenital microcephaly, Flavivirus, Zika virus

## Abstract

**Background:** Zika virus (ZIKV) was first discovered in East Africa in 1947.  ZIKV has caused microcephaly in the Americas, but it is not known whether ZIKV is a cause of microcephaly in East Africa.

**Methods:** We used surveillance data from 11,061 live births at Kilifi County Hospital in coastal Kenya between January 2012 and October 2016 to identify microcephaly cases and conducted a nested case-control study to determine risk factors for microcephaly. Gestational age at birth was estimated based on antenatal ultrasound scanning (‘Scanned cohort’) or last menstrual period (‘LMP cohort’, including births ≥37 weeks’ gestation only). Controls were newborns with head circumference Z scores between >-2 and ≤2 SD that were compared to microcephaly cases in relation to ZIKV exposure and other maternal and newborn factors.

**Results:** Of the 11,061 newborns, 214 (1.9%, 95%CI 1.69, 2.21) had microcephaly. Microcephaly prevalence was 1.0% (95%CI 0.64, 1.70, n=1529) and 2.1% (95%CI 1.81, 2.38, n=9532) in the scanned and LMP cohorts, respectively. After excluding babies <2500 g (n=1199) in the LMP cohort the prevalence was 1.1% (95%CI 0.93, 1.39). Microcephaly showed an association with being born small for gestational age (p<0.001) but not with ZIKV neutralising antibodies (p=0.6) or anti-ZIKV NS1 IgM response (p=0.9). No samples had a ZIKV neutralising antibody titre that was at least fourfold higher than the corresponding dengue virus (DENV) titre. No ZIKV or other flavivirus RNA was detected in cord blood from cases or controls.

**Conclusions:** Microcephaly was prevalent in coastal Kenya, but does not appear to be related to ZIKV exposure; the ZIKV response observed in our study population was largely due to cross-reactive responses to DENV or other related flaviviruses. Further research into potential causes and the clinical consequences of microcephaly in this population is urgently needed.

## Introduction

The recent Zika virus (ZIKV) epidemic in the Americas has focused attention on microcephaly as a major complication of *in-utero* infection and a cause of neurodisability in newborns ^1^. Very little is known about the burden of microcephaly in Africa and, though ZIKV was first discovered in East Africa ^2^ and the Aedes mosquito vector for ZIKV is plentiful, it is not known whether ZIKV is a cause of microcephaly in the region. A cross-sectional survey in 1966-68 found high (52%) ZIKV antibody seroprevalence among children and adults in coastal Kenya ^3^, though antibody cross-reactivity between ZIKV and other flaviviruses in circulation such as dengue virus (DENV) and West Nile virus (WNV) makes the interpretation of these data difficult. Several major flavivirus outbreaks have since occurred in the country ^4– 6^, with various serosurveys indicating ongoing flavivirus exposure ^7, 8^. Notably, high flavivirus antibody seroprevalence was reported amongst pregnant women sampled in 2002-03 in coastal Kenya but the association with birth outcomes was not determined ^9^.

We previously initiated a perinatal and maternal health research programme in coastal Kenya to identify risk factors for: 1) severe morbidity and mortality in mothers and newborns ^10^ and 2) preterm and small for gestational age (SGA) births in the INTERBIO-21 ^st^ Study ^11^. As part of these two studies, we took head circumference measurements and demographic and anthropometric data allowing an estimation of: 1) the prevalence of microcephaly in coastal Kenya; 2) its association with maternal and newborn factors, and 3) its association with flavivirus exposure.

## Methods

### Study population and data collection

This was a population-based, observational, cohort study undertaken at Kilifi County Hospital (KCH) between January 2012 and October 2016. KCH is a rural public county hospital providing comprehensive obstetric care annually to approximately 5,000 women living along the Kenyan coast. All women completed a standardised admission record as part of two studies: an ongoing clinical surveillance study assessing risk factors for severe morbidity and mortality in mothers and newborns ^10^ and the INTERBIO-21 ^st^ Study ^11^. This included socio-demographic information, clinical history including antenatal clinic attendance, clinical findings on admission, delivery details, and maternal and newborn anthropometry. Gestational age was determined either by calculating the difference between the date of delivery and the date of the last reported menstrual period (LMP), including only births ≥37 weeks’ gestation (“LMP cohort”); or by a pregnancy dating ultrasound scan done ≤24 weeks’ gestation for a subset of participants enrolled in the INTERBIO-21 ^st^ Study ^11^, which included preterm and term births, referred to hereafter as the “scanned cohort”.

All newborns had anthropometric measurements (i.e. head circumference, weight and length) taken within 48 hours of birth by nurses and fieldworkers trained as part of the INTERBIO-21 ^st^ Study, which included quarterly refresher training and continual quality control. Anthropometry for the scanned cohort was done in duplicate by two different fieldworkers, and discrepancies resolved by a third measurement. ^11^ Maternal blood for routine and research samples was collected on admission, and umbilical cord blood was collected at delivery. Maternal and cord blood samples were processed, and plasma stored at -80ºC, within 24 hours of collection. All mothers provided written informed consent for use of their biological samples and clinical data. The studies were approved by the Kenya Medical Research Institute (KEMRI) Scientific and Ethics Review Unit (KEMRI SERU # 3296 and 1778).

### Laboratory procedures

***Viral RNA detection.*** For qRT-PCR detection of ZIKV and other flaviviruses, viral RNA was isolated from cord plasma using the QIAamp® Viral RNA kit (Qiagen) according to manufacturer’s instructions. Samples were then screened for ZIKV and other flavivirus RNA using the QuantiFast RT-PCR kit (Qiagen) and published pan-flavivirus (Flavi allS, Flavi all AS2, Flavi all AS4 and Flavi all probe 3 mix) ^12^ and ZIKV-specific primers and probes, Bonn E and Bonn NS1 ^13^, on an ABI 7500 Real Time PCR system. Sequences for all primers can be found in the indicated references. The PCR cycling conditions for the pan-flavivirus assay ^12^ were: 50°C for 20 minutes, 95°C for 15 minutes, followed by 45 cycles comprising 95°C for 15 seconds and 60°C for 1 minute. For ZIKV Bonn E and Bonn NS1 ^13^ the conditions were: 50°C for 20 minutes, 95°C for 15 minutes, followed by 45 cycles comprising 95°C for 15 seconds and 58°C for 1 minute. A cycle threshold value of <40 was used to define positives for all three assays. RNA isolated from ZIKV MR766 strain and a range of other flaviviruses (DENV, WNV and Yellow Fever virus) cultured in Vero E6 cells were used as positive controls, and the PCR mastermix without template used as a negative control in these assays.

***FRNT _90_ assay.*** Cord plasma were screened for antibodies to ZIKV using a ZIKV focus reduction neutralisation test (FRNT _90_) and an in-house IgM ELISA assay against ZIKV NS1 antigen strain MR766. For the FRNT _90_ assay, heat-inactivated cord plasma samples were diluted to 1:20 in 100 µl Dulbecco’s Modified Eagle’s Medium (DMEM) containing 10% fetal calf serum (D10), mixed with an equal volume of D10 containing approximately 100 focus-forming units of ZIKV MR766 strain, and incubated for 1 hour at 37ºC. The virus-plasma mixture was then overlaid onto 96-well flat-bottomed plates containing Vero E6 monolayers at 90% confluency for virus adsorption at 37ºC, 5% CO _2_ for 24 hours. The virus-plasma mixture was aspirated from the wells, 100 µl of D10 was added and the plates incubated for a further 24 hours at 37ºC, 5% CO _2_. Immunostaining was then used to detect virus infection. Briefly, cells were gently washed with phosphate buffered saline (PBS), fixed in 4% paraformaldehyde in PBS for 10 minutes and permeabilized with permeabilization buffer (0.5% Triton X-100 in PBS) for 30 minutes. The plates were blocked in Blocker™ Casein (ThermoFisher) before addition of 0.5 µg/ml of the anti-flavivirus E protein monoclonal antibody 4G2 (Native Antigen, UK, Cat. No AbFLAVENV-4G2) in permeabilization buffer for a 2-hour incubation at 37°C. Following a further series of washes, plates were incubated with a 1:1000 dilution of horseradish peroxidase (HRP)-conjugated goat anti-mouse IgG antibody (Abcam, Cat. No. ab6789) in permeabilization buffer for 1 hour at 37°C, and colour development of foci done by addition of 3,3'-diaminobenzidine (Sigma) substrate for 10 minutes at room temperature. The plates were finally washed, air dried and foci counted using an AID ELISpot reader. Plasma samples that resulted in at least 90% reduction in foci relative to wells incubated with virus only were considered flavivirus seropositive. Antibody titres against the ZIKV MR766 strain, and against a local DENV-2 isolate obtained from a patient in coastal Kenya, were then estimated for seropositive samples using the FRNT _90_ assay on twofold serial plasma dilutions. FRNT _90_ antibody titres were calculated using the Reed and Muench method ^14^.

***ZIKV NS1 IgM ELISA.*** For the IgM assay, 96-well flat-bottomed plates were first coated with 1 µg/ml of the ZIKV NS1 antigen (Native Antigen, UK, Cat. No. ZIKV-NS1) at room temperature overnight, then washed in wash buffer (0.05% Tween in PBS) and blocked with Blocker™ Casein (ThermoFisher) for 1 hour. Cord plasma were diluted 1:400 in Blocker™ Casein, added to plates in duplicate and incubated for 2 hours at room temperature. After a further series of washes, a 1:5000 dilution of HRP-conjugated goat anti-human IgM antibody (KPL, Cat. No. 074-1003) in wash buffer was added to plates, incubated for 1 hour, washed and OPD substrate (Sigma) added for colour development for 15 minutes. Plates were read on a Biotek ELISA reader at a wavelength of 492 nm and optical density values for each sample acquired for analysis. Plasma from coastal Kenya residents with previous PCR-confirmed DENV infection ^15^ were used as positive controls, while plasma from two European individuals and a pool of cord plasma from 10 neonates without detectable responses to the ZIKV NS1 antigen were used as negative controls. IgM ratios, defined as the ratio between mean sample OD and the mean OD of the negative controls, were then obtained and seropositivity defined as an IgM ratio of >3 as done by others ^16^.

### Statistical analysis

Based on World Health Organization recommendations ^14^, microcephaly (cases) was defined as a birth head circumference (HC) Z score < -3 SD from the mean for gestational age and sex using INTERGROWTH-21 ^st^ (IG21) newborn size reference charts for births <33 weeks’ gestation ^15^, and standards for births ≥33 weeks’ gestation ^16^. Controls were defined as newborns with HC Z scores between > -2 and ≤ 2 SD. Univariable logistic regression models were used to estimate associations between microcephaly and maternal or newborn variables hypothesised to be putative risk factors (22 variables tested; Table 1). A nominal two-sided p value was calculated as <0.002 (i.e. 0.05 divided by 22 for the number of covariates) following Bonferroni adjustment for multiple testing. Variables reaching the nominal p<0.002 were then included in a multivariable logistic regression model and their adjusted association with microcephaly estimated. All logistic regression analyses were performed using pooled data from the scanned and LMP cohorts, respectively. To assess the distribution of variables between the two cohorts stratified analyses were performed using χ ^2^ tests (for categorical variables) and Mann-Whitney U-tests (for continuous variables). All analyses were carried out in Stata™ version 15 with two-sided p-values reported.

**Table 1.** Association between maternal and newborn co-factors and microcephaly. The total number and prevalence of cases in the final case-control dataset, stratified by categories of neonatal and maternal variables, are shown. Data from the scanned and LMP cohorts are pooled in these analyses, but cohort-specific frequencies are shown in Table 3. Crude odds ratios (OR), 95% confidence intervals (CI) and P values from univariable logistic regression models estimating associations with microcephaly with each variable in turn are shown. The reference population in each of the models is assigned a value of 1. The total number of newborns included in each analysis varies due to missing data for some variables. *Systolic or diastolic blood pressure >140 or >90 mmHg, respectively.

Covariate	Categories	n/N cases (%)	Crude OR (95% CI)	P value
*Newborn factors*				
Sex	Male	69/4794 (1.4%)	1	
	Female	42/4303 (1.0%)	0.67 (0.46, 0.99)	0.05
Small for gestational age (SGA)	Normal	61/7331 (0.8%)	1	
	SGA	50/1766 (2.8%)	3.47 (2.38, 5.07)	<0.001
Type of birth	Singleton	106/8879 (1.2%)	1	
	Multifetal	5/218 (2.3%)	1.9 (0.80, 4.75)	0.14
Year of birth	2012	17/1381 (1.2%)	1	
	2013	28/1499 (1.9%)	1.53 (0.83, 2.80)	0.17
	2014	23/2379 (1.0%)	0.78 (0.42, 1.47)	0.45
	2015	23/2043 (1.1%)	0.91 (0.49, 1.72)	0.78
	2016	20/1795 (1.1%)	0.90 (0.47, 1.73)	0.76
Season	January – March	24/2245 (1.1%)	1	
	April – June	41/2742 (1.5%)	1.40 (0.85, 2.33)	0.19
	July – September	22/2240 (1.0%)	0.92 (0.51, 1.64)	0.77
	October – December	24/1870 (1.3%)	1.20 (0.68, 2.13)	0.52
*Maternal factors*				
*Sociodemographic factors*				
Maternal age	<20 years	17/1339 (1.3%)	1	
	20 to 35 years	87/6966 (1.2%)	0.98 (0.58, 1.66)	0.95
	>35 years	7/783 (0.9%)	0.70 (0.29, 1.70)	0.43
Marital status	Married	99/8329 (1.2%)	1	
	Unmarried	10/665 (1.5%)	1.27 (0.66, 2.44)	0.47
Education level	Secondary or more	21/2627 (0.8%)	1	
	Primary school	73/5253 (1.4%)	1.75 (1.07, 2.85)	0.02
	None	13/1010 (1.3)	1.62 (0.81, 3.24)	0.17
Residence	Other	70/5893 (1.2%)	1	
	Kilifi township	41/3176 (1.3%)	1.09 (0.74, 1.60)	0.67
Type of house	Stone wall	45/4702 (1.0%)	1	
	Mud wall	63/4328 (1.5%)	1.53 (1.04, 2.25)	0.03
Obstetric history				
Parity	Primigravida	41/3302 (1.2%)	1	
	Multigravida	70/5738 (1.2%)	0.98 (0.67, 1.45)	0.93
Antenatal care attendance	≥4 visits	69/5887 (1.2%)	1	
	0 to 3 visits	42/3434 (1.3%)	1.05 (0.72, 1.55)	0.79
*Medication during pregnancy*				
Folic acid supplements	Yes	102/8292 (1.2%)	1	
	No	9/795 (1.1%)	0.92 (0.46, 1.82)	0.81
Malaria prophylaxis	≥3 doses	61/5166 (1.2%)	1	
	1 to 2 doses	47/3070 (1.5%)	1.30 (0.89, 1.91)	0.18
	None	3/689 (0.4%)	0.37 (0.11, 1.17)	0.09
Tetanus vaccination	Yes	95/7706 (1.2%)	1	
	No	13/1137 (1.1%)	0.93 (0.52, 1.66)	0.80
*Maternal co-morbidities and infections*				
Mid-upper arm circumference (MUAC)	Normal (23–30cm)	76/6490 (1.2%)	1	
	Low (<23cm)	13/990 (1.3%)	1.12 (0.62, 2.03)	0.70
	High (≥30cm)	9/997 (0.9%)	0.77 (0.38, 1.54)	0.46
Hypertension in pregnancy *	No	92/7623(1.2%)	1	
	Yes	9/540 (1.7%)	1.39 (0.70, 2.77)	0.35
HIV status	Negative	107/8605 (1.2%)	1	
	Positive	5/365 (1.1%)	0.88 (0.32, 2.40)	0.80
Maternal anaemia	No	21/2321 (0.9%)	1	
	Yes	66/5436 (1.2%)	1.35 (0.82, 2.20)	0.24
VDRL (syphilis test)	Negative	95/8014 (1.2%)	1	
	Positive	1/45 (2.2%)	1.89 (0.26, 13.89)	0.53
*Other risk exposures*				
Substance use	No	108/8855 (1.2%)	1	
	Yes	3/232 (1.3%)	1.06 (0.33, 3.37)	0.92
Contact with cattle	No	101/8225 (1.2%)	1	
	Yes	7/463 (1.5%)	1.23 (0.57, 2.67)	0.59

## Results

### Prevalence of microcephaly

**Figure 1.**
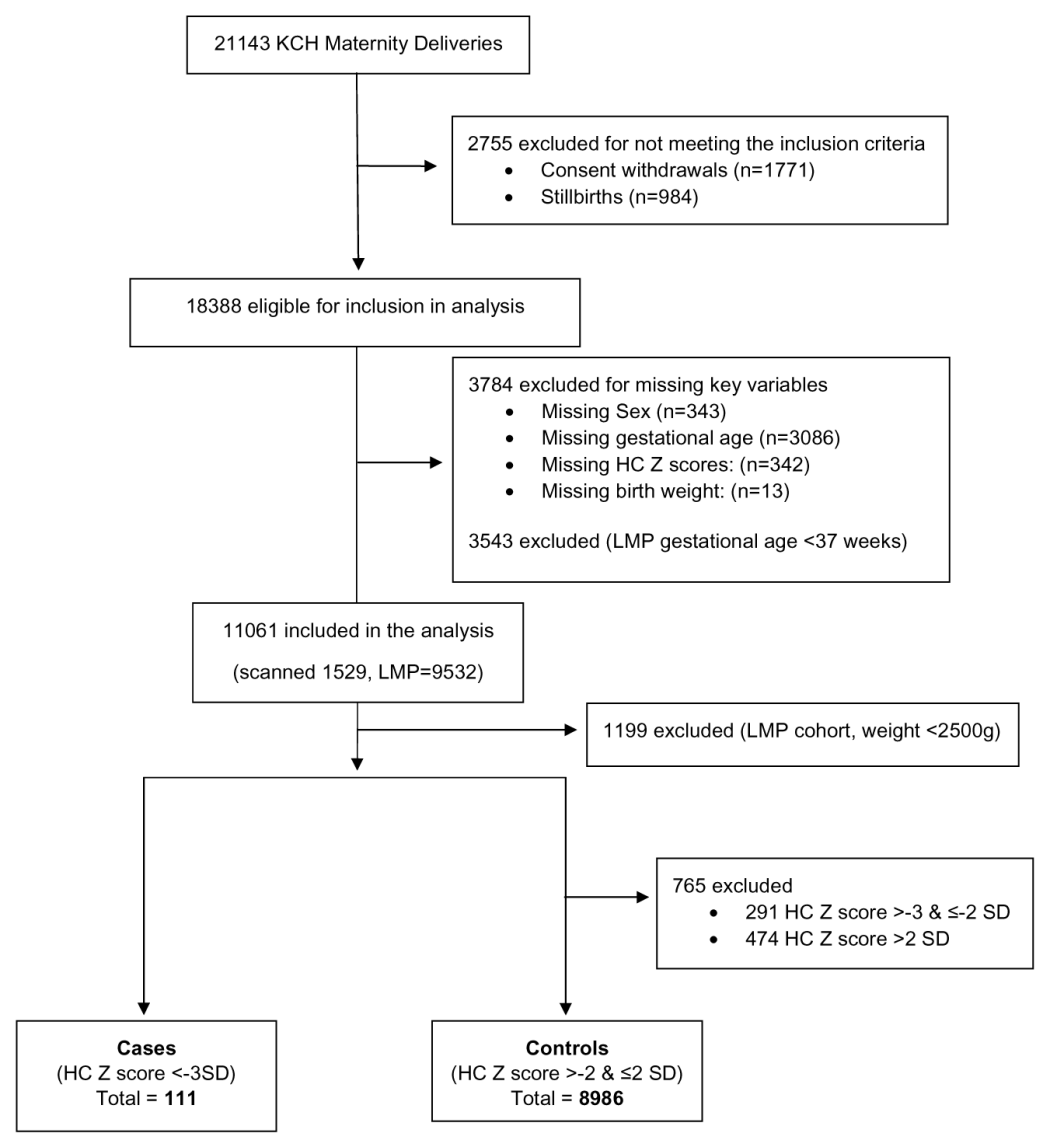
Study participants flow diagram.

Between January 2012 and October 2016 there were 21,143 births at KCH. We excluded stillbirths (n=984), consent withdrawals (n=1771), births with missing key variables (sex, gestational age, HC Z scores and birth weight; n=3784) and preterm newborns for the LMP cohort only (n=3543). We included 11061 live births in the main analysis ( Figure 1). The mean gestational age of newborns in the scanned cohort was 38.6 weeks (95% CI 38.44, 38.66) and 39.3 weeks (95% CI 39.26, 39.32) for newborns in the LMP cohort.

**Figure 2.**
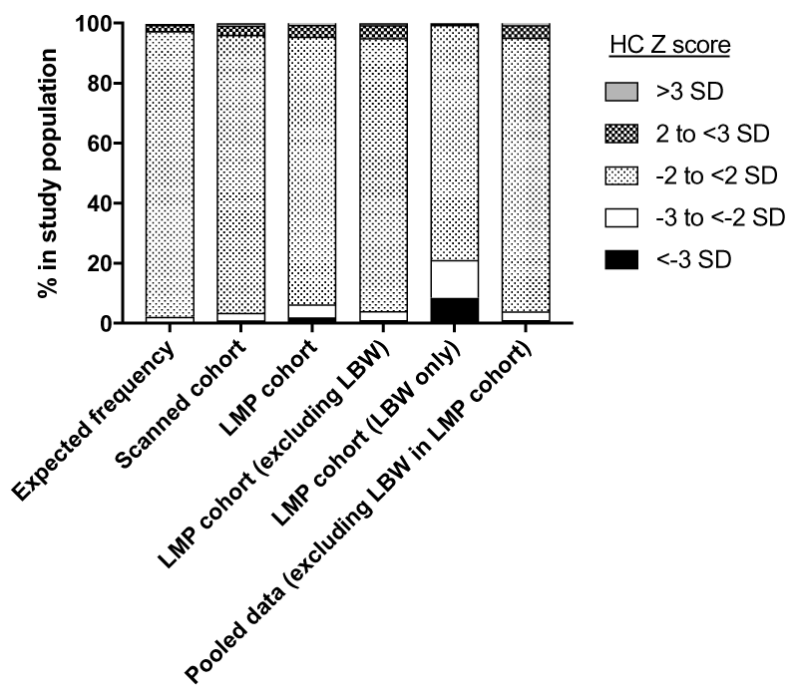
Distribution of head circumference (HC) Z scores in the study population. The distribution of HC Z scores in the study population, measured as described in the *Methods* section, are shown. For comparison, the expected frequencies in a normal distribution are shown. For the LMP cohort distributions are shown for the full cohort, after exclusion of newborns with low birth weight (LBW; <2500 g), or for LBW newborns only. Distributions for the final analysis dataset (‘Pooled data [excluding LBW in LMP cohort]) are shown for comparison.

There was an excess frequency of births with HC Z scores below -3 SD in the study population when compared to the expected normal distribution ( Figure 2). However, the observed frequency of births with HC Z scores between -3 and <-2 SD was similar to the expected normal distribution ( Figure 2).

A total of 16 (1.0%) of the 1529 newborns in the scanned cohort, and 198 (2.1%) of the 9532 newborns in the LMP cohort had a HC Z score <-3 SD. We hypothesised that the higher case prevalence in the LMP cohort was due to the known inaccuracy of LMP in estimating gestational age as compared to ultrasound scans, leading to preterm births being classified as term, in turn resulting in a lower Z score than would have been assigned had the gestational age been known accurately ^14^. Indeed, among 1399 births (1253 term, 146 preterm) in the scanned cohort (used as gold standard) that also had corresponding LMP gestation ages, LMP gestation would have misclassified 9.3% (117/1253) of the term births as preterm births, and 39.7% (58/146) of preterms as term births. Therefore, to enhance the specificity of our case definition for associations, we excluded newborns with low birth weight (<2500 g, as per WHO guidelines) from the analysis of the LMP cohort. When this exclusion was applied the prevalence of microcephaly in the LMP cohort was 1.1% (95% CI 0.93, 1.39) among newborns weighing ≥2500 g (n=8333) and 8.6% (95% CI 7.13, 10.32) among those weighing <2500 g (n=1199), a difference that was statistically significant (χ ^2^=286.05, p<0.001; Fig. 2). In contrast, low birth weight showed no association with microcephaly in the scanned cohort (χ ^2^=0.40, p=0.5). All further analyses on the LMP cohort were therefore restricted to newborns weighing ≥2500 g at birth, which when pooled with the scanned cohort gave a case prevalence of 1.1% (95% CI 0.93, 1.35).

### Associations between microcephaly and maternal and newborn factors

To identify potential risk factors for microcephaly, we used a nested case-control approach whereby cases (n=111) were compared to controls (newborns with HC Z score > -2 and ≤ 2 SD, n=8986) with respect to various maternal and newborn variables by logistic regression ( Table 1 and Table 3). A strong association was observed between microcephaly and being born small for gestational age (SGA), defined as birth weight <10 ^th^ centile for gestational age and sex on IG21 charts (OR=3.47, 95% CI 2.38, 5.07, p<0.001). Maternal nutritional status, anaemia, HIV status, parity, receipt of interventions provided in the antenatal clinic and all other newborn and maternal factors tested showed no significant association with microcephaly.

### Associations between microcephaly and flavivirus exposure

We used qRT-PCR and serological assays to investigate whether flavivirus exposure was associated with microcephaly in our dataset. To test for recent exposure to flavivirus, we measured IgM antibody responses against ZIKV NS1 antigen in cord plasma from 94 cases with available samples and 864 controls matched by year of birth. Overall IgM seropositivity against ZIKV NS1 was 2.4% (95% CI 1.60, 3.59), though this was strongly confounded by cross-reactive responses to DENV since sera from 25 patients from coastal Kenya with lab-confirmed dengue infection ^17, 18^ were all seropositive on the ZIKV NS1 IgM assay. No association was evident between the ZIKV NS1 IgM response and microcephaly ( Table 2). Furthermore, no ZIKV or other flavivirus RNA could be detected in cord plasma from the 94 cases or from a random selection of controls (n=471).

**Table 2.** Associations between flavivirus serology and microcephaly. Prevalence of anti-ZIKV antibody responses as measured by FRNT _90_ assay and IgM ELISA is shown for cases and controls. Odds ratios, 95% confidence intervals and p value for the association with microcephaly are shown.

		Seropositivity n/N (%)	Crude OR (95% CI)	P
ZIKV NS1 IgM assay	Controls	21/864 (2.4%)	1	
	Cases	2/94 (2.1%)	0.87 (0.20, 3.78)	0.86
ZIKV FRNT90 assay	Controls	61/755 (8.1%)	1	
	Cases	7/71 (9.7%)	1.24 (0.55, 2.83)	0.60

**Table 3.** Stratified analyses estimating associations between microcephaly and maternal and newborn co-factors in each cohort. Univariate analyses assessing the relationship between maternal and newborn factors and microcephaly in each cohort are shown. For each cohort the frequency of cases is shown, and χ ^2^ test used for all analyses. When all variables that were statistically significant at p<0.05 in any of the cohorts (indicated by *) were included in a multivariable logistic regression model, only SGA maintained an association with microcephaly (adjusted OR=3.41, 95% CI 2.30, 5.06, p<0.001).

	LMP cohort			Scanned cohort	
Covariate	Categories	n/N cases (%)	P value	n/N cases (%)	P value
**A) Newborn factors**					
Sex	Male	58/4047 (1.4%)	0.10	11/747 (1.5%)	0.18
	Female	37/3618 (1.0%)		5/685 (0.7%)	
Small for gestational age (SGA) *	Normal	51/6184 (0.8%)	<0.001	10/1147 (0.9%)	0.08
	SGA	44/1481 (3.0%)		6/285 (2.1%)	
Type of birth *	Singleton	92/7472 (1.2%)	0.69	14/1393 (1.0%)	0.001
	Multifetal	3/193 (1.5%)		2/25 (8.0%)	
Year of birth	2012	17/1312 (1.3%)	0.05	0/69 (0)	0.75
	2013	25/1225 (2.0%)		3/274 (1.1%)	
	2014	14/1712 (0.8%)		9/667 (1.3%)	
	2015	19/1621 (1.2%)		4/422 (0.9%)	
	2016	20/1795 (1.1%)		No data	
^&^Season	January - March	21/1835 (1.1%)	0.33	3/410 (0.7%)	0.18
	April - June	34/2379 (1.4%)		7/363 (1.9%)	
	July - September	17/1899 (0.9%)		5/341 (1.5%)	
	October - December	23/1552 (1.5%)		1/318 (0.3%)	
**B) Maternal factors**					
**Sociodemographic factors**					
Maternal age	<20 years	14/1164 (1.2%)	0.49	3/175 (1.7%)	0.59
	20 to 35 years	76/5835 (1.3%)		11/1131 (1.0%)	
	>35 years	5/659 (0.7%)		2/124 (1.6%)	
Marital status *	Married	87/6999 (1.2%)	0.68	12/1330 (0.9%)	0.002
	Unmarried	6/575 (1.2%)		4/90 (4.4%)	
Education level	Secondary or more	20/2221 (0.9%)	0.22	1/406 (0.2%)	0.15
	Primary school	62/4443 (1.4%)		11/810 (1.4%)	
	None	10/832 (1.2%)		3/178 (1.7%)	
Residence	Other	60/5293 (1.1%)	0.19	10/600 (1.7%)	0.09
	Kilifi township	35/2346 (1.5%)		6/830 (0.7%)	
Type of house *	Stone wall	36/3902 (0.9%)	0.02	9/800 (1.1%)	0.99
	Mud wall	56/3708 (1.5%)		7/620 (1.1%)	
**Obstetric history**					
Parity	Primigravida	39/2814 (1.4%)	0.40	2/488 (0.4%)	0.06
	Multigravida	56/4812 (1.2%)		14/926 (1.5%)	
**Antenatal care**					
Antenatal care attendance	≥4 visits	57/4501 (1.3%)	0.87	12/1221 (1.0%)	0.22
	0 to 3 visits	38/3102 (1.2%)		4/203 (2.0%)	
**Medication during pregnancy**					
Folic acid supplements	Yes	86/6947 (1.2%)	0.95	16/1345 (1.2%)	0.32
	No	9/713 (1.3%)		0/82 (0)	
Malaria prophylaxis	≥3 doses	53/4106 (1.3%)	0.07	8/1060 (0.7%)	0.05
	1 to 2 doses	40/2785 (1.4%)		7/285 (2.5%)	
	None	2/632 (0.3%)		1/57 (1.7%)	
Tetanus vaccination	Yes	79/6456 (1.2%)	0.82	16/1250 (1.3%)	0.17
	No	13/994 (1.3%)		0/143 (0)	
**Maternal co-morbidities and infections**					
Mid-upper arm circumference (MUAC)	Normal (23-30cm)	65/5417 (1.2%)	0.85	11/1073 (1.0%)	0.22
	Low (<23cm)	9/822 (1.1%)		4/168 (2.4%)	
	High (≥30cm)	8/812 (1.0%)		1/185 (0.5%)	
Hypertension in pregnancy	No	77/6296 (1.2%)	0.31	15/1327 (1.1%)	0.996
	Yes	8/452 (1.8%)		1/88 (1.1%)	
HIV status	Negative	91/7277 (1.1%)	0.78	16/1328 (1.2%)	0.30
	Positive	4/278 (1.4%)		0/87 (0)	
Maternal anaemia	No	19/1885 (1.0%)	0.46	2/436 (0.5%)	0.21
	Yes	56/4570 (1.2%)		10/866 (1.1%)	
VDRL (syphilis test)	Negative	79/6732 (1.2%)	0.34	16/1282 (1.2%)	0.71
	Positive	1/34 (2.9%)		0/11 (0)	
**Other risk exposures**					
Substance use	No	93/7459 (1.2%)	0.75	15/1396 (1.1%)	0.26
	Yes	2/201 (1.0%)		1/31 (3.2%)	
Contact with cattle	No	87/6907 (1.3%)	0.99	14/1318 (1.1%)	0.13
	Yes	5/399 (1.2%)		2/64 (3.1%)	

**Figure 3.**
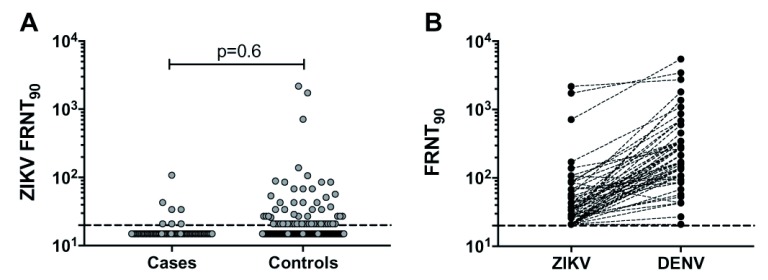
DENV and ZIKV neutralising antibody titres in cord plasma. FRNT _90_ antibody titres measured against ZIKV MR766 strain in cord plasma from cases and controls are shown in ( **A**), including p value from statistical comparison using the Mann-Whitney U test. For ZIKV FRNT _90_ seropositive samples (n=68) the corresponding FRNT _90_ antibody titres against a local DENV-2 isolate are shown in ( **B**). The dashed line represents the assay limit of detection.

Of the 94 cases with samples available, 71 had sufficient cord plasma material for ZIKV FRNT _90_ assay. For the FRNT _90_ assay, each of the 71 cases were matched to at least 10 controls by year of birth (n=755). The overall ZIKV FRNT _90_ antibody prevalence in the study population was 8.2% (95% CI 6.54, 10.32), but this showed no association with microcephaly ( Table 2 and Figure 3).

To characterise the ZIKV FRNT _90_ response further we measured FRNT _90_ antibody levels of the ZIKV FRNT _90_ seropositive cord plasma (7 cases, 61 controls) against a local DENV-2 isolate and compared these to the corresponding ZIKV FRNT _90_ titres. All ZIKV seropositive samples had antibody against DENV, with DENV FRNT _90_ titres for most samples (43 of the 68 ZIKV seropositive samples) being at least fourfold higher than the corresponding ZIKV FRNT _90_ titres ( Figure 3). No sample had a ZIKV FRNT _90_ titre that was at least fourfold higher than that for DENV suggesting that the ZIKV response observed in our study population was largely due to cross-reactive responses to DENV or other related flaviviruses. Raw data used in these analyses are available as *Underlying data* ^19^.

## Discussion

This study set out to estimate the prevalence of microcephaly in coastal Kenya in a cohort of babies born in a rural public county hospital using data from two prospective studies that estimated gestational age clinically and using ultrasound, respectively. We then sought to identify risk factors for microcephaly using a nested case-control design ^10^. We found a prevalence of 1 to 2%, which given the 0.1% expected prevalence (<-3 SD in the reference populations) suggests a high, previously unrecognised, burden of microcephaly in this region. Using logistic regression we found that newborns with microcephaly were more likely to be born SGA. Finally, we did not detect any ZIKV or flavivirus RNA among cases or controls; nor differences in anti-ZIKV antibody responses between cases and controls.

A similarly high prevalence of microcephaly has been observed in Nigeria where approximately 1.9% of infants had a HC Z score <-3 SD ^20^. In comparison, the average pre-ZIKV epidemic prevalence of microcephaly in 55 hospitals in the Americas was estimated at 0.04% (95% CI 0.041, 0.049) ^21^, while the prevalence among 24 European Surveillance of Congenital Anomalies registries ranged between 0.004% and 0.04% ^22^. During the ZIKV epidemic the overall microcephaly prevalence (HC Z score <-3 SD) in Pernambuco, one of the most severely affected states in Brazil, was estimated at 0.15% (95% CI 0.12, 0.17) ^23^, almost ten times lower than that observed in our dataset.

Definitions of microcephaly are complex and confounded by prematurity. Could we have overestimated the prevalence? We showed that use of the LMP led to an overestimate of gestational age compared with the gold-standard method of ultrasound scans in early pregnancy, which may have led to an overestimate in the prevalence of microcephaly. Furthermore, errors in measuring head circumference could lead to misclassification. However, when we restricted analysis to the scanned cohort, in which gestational ages were calculated by high quality ultrasound scans in early pregnancy, and where two anthropometrists confirmed head circumferences, the prevalence of microcephaly remained high. Furthermore, even after restricting analysis in the LMP cohort to newborns with birth weight ≥2500 g, thus excluding all low birth weight (and hence most preterm births), the prevalence of microcephaly remained high.

We undertook detailed serological testing in cases and controls and did not find any ZIKV or flavivirus RNA. In addition, we showed no differences in anti-ZIKV FRNT _90_ or IgM antibody response between cases and controls. Notably, all the ZIKV FRNT _90_ seropositive samples were also seropositive for DENV. Comparison of FRNT _90_ titres is conventionally used to infer specific flavivirus exposure ^24^; if the detected responses were due to recent ZIKV exposure we would expect ZIKV FRNT _90_ titres to be at least fourfold higher than the DENV FRNT _90_ titres in the corresponding sample(s) ^24^. However, no sample had a ZIKV FRNT _90_ titre that was fourfold higher than the corresponding DENV FRNT _90_ titre. In fact, for more than half (63%) of the seropositive samples DENV FRNT _90_ titres were at least fourfold higher than ZIKV FRNT _90_ titres. Furthermore, the lack of seasonality in the risk of microcephaly, lack of trend by calendar year, and absence of any difference by urban/rural residence suggest it is unlikely that a vector borne or respiratory infection, including ZIKV, is the cause of microcephaly in our setting. The only strongly significant risk factor for microcephaly in our population was SGA; this sub-group accounted for 45% of all cases.

It is possible that we missed the viraemia in cord blood as a result of infection occurring earlier in pregnancy, as is common for ZIKV-associated microcephaly ^1, 25^. Sample collection in this study was only done at the time of delivery ^10^. However, if ZIKV was a significant cause of microcephaly we would expect an increase in IgM levels to ZIKV in cord blood, as reported elsewhere among children with microcephaly secondary to ZIKV ^26– 29^. Furthermore, although molecular evidence of infection is not common among newborns with ZIKV-induced microcephaly, we would expect at least some to have prolonged ZIKV viraemia if this were a common cause. The complete absence of any molecular evidence of ZIKV in cases and controls, including in newborns with measurable cord blood anti-ZIKV FRNT _90_ and IgM antibody responses, leads us to believe that the anti-ZIKV response detected in our study is cross-reactive to other flaviviruses. This hypothesis is supported by the high seroprevalence of DENV antibodies measured FRNT _90_ in this study and by other methods in previous studies in coastal Kenya ^7– 9, 30^. Other potential infectious and non-infectious causes ^31, 32^, including genetic, nutritional and environmental factors, also warrant further investigation and may underlie the associations observed between microcephaly and SGA newborns (as observed by others ^29^).

In addition to using LMP in a subgroup to define gestational age, this study had other limitations. The study population only included births at KCH, and hence will have missed births occurring at home or in other local health facilities. However, we have important data from our uniquely detailed demographic surveillance system which indicates that approximately 40% of all births in the hospital catchment area occur at KCH.

This study has allowed the first estimation of the risk of congenital microcephaly in coastal Kenya. A 1-2% prevalence of microcephaly may impose a public health burden depending on the clinical outcomes associated. Future prospective studies to characterise and determine post-discharge mortality, neurocognitive outcomes and aetiology of microcephaly in the region are a priority.

## Data availability

### Underlying data

Harvard Dataverse: Replication Data for: Congenital Microcephaly Unrelated to Flavivirus Exposure in Coastal Kenya. https://doi.org/10.7910/DVN/4EB9PG ^19^.

This project contains the following underlying data:

- microcephaly_dataverse_v1 (dataset containing demographic information, anthropometric measures and results of lab assays for participants included in the study).
- GWarimwe_Microcephaly_Codebook (contains variable description and value labels).

### Extended data

Harvard Dataverse: Replication Data for: Congenital Microcephaly Unrelated to Flavivirus Exposure in Coastal Kenya. https://doi.org/10.7910/DVN/4EB9PG ^19^.

This project contains the following extended data:

- GWarimwe_Microcephaly_missing_data_summary (summary of missing data for analysed variables).
- GWarimwe_Microcephaly_readme (readme file).
- microcephaly_dataverse_final (STATA analysis code used for data analysis presented in this article).

Data are available under the terms of the Creative Commons Attribution 4.0 International license (CC-BY 4.0).

## References

[1] Petersen LR Jamieson DJ Powers AM: Zika Virus. *N Engl J Med.* 2016;374(16):1552–63. 10.1056/NEJMra1602113 27028561

[2] Dick GW: Zika virus. II. Pathogenicity and physical properties. *Trans R Soc Trop Med Hyg.* 1952;46(5):521–34. 10.1016/0035-9203(52)90043-6 12995441

[3] Geser A Henderson BE Christensen S: A multipurpose serological survey in Kenya. 2. Results of arbovirus serological tests. *Bull World Health Organ.* 1970;43(4):539–52. 5313066 PMC2427766

[4] Johnson BK Musoke S Ocheng D: Dengue-2 virus in Kenya. *Lancet.* 1982;2(8291):208–9. 10.1016/s0140-6736(82)91047-9 6123900

[5] Vu DM Mutai N Heath CJ: Unrecognized Dengue Virus Infections in Children, Western Kenya, 2014-2015. *Emerg Infect Dis.* 2017;23(11):1915–7. 10.3201/eid2311.170807 29048283 PMC5652413

[6] Ellis BR Barrett AD: The enigma of yellow fever in East Africa. *Rev Med Virol.* 2008;18(5):331–46. 10.1002/rmv.584 18615782

[7] Mease LE Coldren RL Musila LA: Seroprevalence and distribution of arboviral infections among rural Kenyan adults: a cross-sectional study. *Virol J.* 2011;8:371. 10.1186/1743-422X-8-371 21794131 PMC3161961

[8] Ochieng C Ahenda P Vittor AY: Seroprevalence of Infections with Dengue, Rift Valley Fever and Chikungunya Viruses in Kenya, 2007. *PLoS One.* 2015;10(7):e0132645. 10.1371/journal.pone.0132645 26177451 PMC4503415

[9] Sutherland LJ Cash AA Huang YJ: Serologic evidence of arboviral infections among humans in Kenya. *Am J Trop Med Hyg.* 2011;85(1):158–61. 10.4269/ajtmh.2011.10-0203 21734142 PMC3122361

[10] Seale AC Barsosio HC Koech AC: Embedding surveillance into clinical care to detect serious adverse events in pregnancy. *Vaccine.* 2015;33(47):6466–8. 10.1016/j.vaccine.2015.07.086 26254977 PMC4817214

[11] Kennedy SH Victora CG Craik R: Deep clinical and biological phenotyping of the preterm birth and small for gestational age syndromes: The INTERBIO-21 ^st^ Newborn Case-Control Study protocol [version 2; peer review: 2 approved]. *Gates Open Res.* 2019;2:49. 10.12688/gatesopenres.12869.2 31172050 PMC6545521

[12] Patel P Landt O Kaiser M: Development of one-step quantitative reverse transcription PCR for the rapid detection of flaviviruses. *Virol J.* 2013;10:58. 10.1186/1743-422X-10-58 23410000 PMC3616844

[13] Corman VM Rasche A Baronti C: Assay optimization for molecular detection of Zika virus. *Bull World Health Organ.* 2016;94(12):880–92. 10.2471/BLT.16.175950 27994281 PMC5153932

[14] World Health Organization: Screening, assessment and management of neonates and infants with complications associated with Zika virus exposure in utero.WHO/ZIKV/MOC/163/Rev3.2016 Reference Source

[15] Villar J Giuliani F Fenton TR: INTERGROWTH-21st very preterm size at birth reference charts. *Lancet.* 2016;387(10021):844–5. 10.1016/S0140-6736(16)00384-6 26898853

[16] Villar J Cheikh Ismail L Victora CG: International standards for newborn weight, length, and head circumference by gestational age and sex: the Newborn Cross-Sectional Study of the INTERGROWTH-21 ^st^ Project. *Lancet.* 2014;384(9946):857–68. 10.1016/S0140-6736(14)60932-6 25209487

[17] Ngoi CN Price MA Fields B: Dengue and Chikungunya Virus Infections among Young Febrile Adults Evaluated for Acute HIV-1 Infection in Coastal Kenya. *PLoS One.* 2016;11(12):e0167508. 10.1371/journal.pone.0167508 27942016 PMC5152832

[18] Kamau E Agoti CN Ngoi JM: Complete Genome Sequences of Dengue Virus Type 2 Strains from Kilifi, Kenya. *Microbiol Resour Announc.* 2019;8(4):pii: e01566–18. 10.1128/MRA.01566-18 30701251 PMC6346200

[19] Barsosio HC Gitonga JN Karanja HK: Replication Data for: Congenital Microcephaly Unrelated to Flavivirus Exposure in Coastal Kenya. Harvard Dataverse, V2.2019 10.7910/DVN/4EB9PG PMC7059837 32175480

[20] Olusanya BO: Full-term newborns with congenital microcephaly and macrocephaly in Southwest Nigeria. *Int Health.* 2012;4(2):128–34. 10.1016/j.inhe.2011.12.006 24029151

[21] Orioli IM Dolk H Lopez-Camelo JS: Prevalence and clinical profile of microcephaly in South America pre-Zika, 2005-14: prevalence and case-control study. *BMJ.* 2017;359:j5018. 10.1136/bmj.j5018 29162597 PMC5696624

[22] Morris JK Rankin J Garne E: Prevalence of microcephaly in Europe: population based study. *BMJ.* 2016;354:i4721. 10.1136/bmj.i4721 27623840 PMC5021822

[23] Kleber de Oliveira W Cortez-Escalante J De Oliveira WT: Increase in Reported Prevalence of Microcephaly in Infants Born to Women Living in Areas with Confirmed Zika Virus Transmission During the First Trimester of Pregnancy - Brazil, 2015. *MMWR Morb Mortal Wkly Rep.* 2016;65(9):242–7. 10.15585/mmwr.mm6509e2 26963593

[24] World Health Organization: Laboratory testing for Zika virus infection.WHO/ZIKV/LAB/161.2016 Reference Source

[25] Cauchemez S Besnard M Bompard P: Association between Zika virus and microcephaly in French Polynesia, 2013-15: a retrospective study. *Lancet.* 2016;387(10033):2125–32. 10.1016/S0140-6736(16)00651-6 26993883 PMC4909533

[26] Cordeiro MT Pena LJ Brito CA: Positive IgM for Zika virus in the cerebrospinal fluid of 30 neonates with microcephaly in Brazil. *Lancet.* 2016;387(10030):1811–2. 10.1016/S0140-6736(16)30253-7 27103126

[27] Cordeiro MT Brito CA Pena LJ: Results of a Zika Virus (ZIKV) Immunoglobulin M-Specific Diagnostic Assay Are Highly Correlated With Detection of Neutralizing Anti-ZIKV Antibodies in Neonates With Congenital Disease. *J Infect Dis.* 2016;214(12):1897–904. 10.1093/infdis/jiw477 27923950

[28] Lanciotti RS Kosoy OL Laven JJ: Genetic and serologic properties of Zika virus associated with an epidemic, Yap State, Micronesia, 2007. *Emerg Infect Dis.* 2008;14(8):1232–9. 10.3201/eid1408.080287 18680646 PMC2600394

[29] de Araújo TVB Ximenes RAA Miranda-Filho DB: Association between microcephaly, Zika virus infection, and other risk factors in Brazil: final report of a case-control study. *Lancet Infect Dis.* 2018;18(3):328–36. 10.1016/S1473-3099(17)30727-2 29242091 PMC7617036

[30] Geser A Christensen S Thorup I: A multipurpose serological survey in Kenya. 1. Survey methods and progress of field work. *Bull World Health Organ.* 1970;43(4):521–37. 5313065 PMC2427784

[31] Devakumar D Bamford A Ferreira MU: Infectious causes of microcephaly: epidemiology, pathogenesis, diagnosis, and management. *Lancet Infect Dis.* 2018;18(1):e1–e13. 10.1016/S1473-3099(17)30398-5 28844634

[32] Nawathe A Doherty J Pandya P: Fetal microcephaly. *BMJ.* 2018;361:k2232. 10.1136/bmj.k2232 29866660

