# Supplementary material for: Congenital microcephaly unrelated to flavivirus exposure in coastal Kenya
Source: Wellcome Open Res. 2019 Nov 15;4:179. [Version 1] doi: 10.12688/wellcomeopenres.15568.1 (PMC7059837; doi:10.12688/wellcomeopenres.15568.1)
Supplement: Peer review history

# Peer review: Reviewer response for version 1

**DOI:** 10.21956/wellcomeopenres.17049.r37360

**Published:** 2020-03-02

**Authors:** Helen Dolk

**Affiliations:** 1 Maternal Fetal and Infant Research Centre, Institute of Nursing and Health Research, Ulster University, Newtownabbey, UK

Barsosio *et al.* have started to address in this paper a gap in the knowledge of both microcephaly and Zika virus in Africa. They investigate the prevalence of microcephaly in a coastal Kenyan population, its association with a range of maternal and newborn factors, whether Zika is circulating in this population, and, if so, whether there is evidence that Zika is causing microcephaly.

 The data used for this study were not specifically designed to answer these questions, but the authors try to address the data quality problems as well as possible.

 The accuracy of classification of babies at birth as microcephalic or not depends on both accuracy of estimation of gestational age and of head circumference. The authors concentrate on the former source of inaccuracy, but it would be interesting to know also (if the data were recorded) the extent of discordant head circumferences in the smaller “scanned cohort” where duplicate measures were made. In addition, it would be informative to add a Figure showing the head circumferences and gestational ages of the cases <-3SD (classified as microcephaly) in the scanned cohort, shown against the Intergrowth chart.

 In clinical practice, one would not expect a clinical diagnosis of microcephaly to be made without further neonatal measurements (including making sure any head molding at birth has resolved), so a degree of misclassification will inevitably remain, with a possible impact on the estimated prevalence, and diluting associations with risk factors.

 The microcephaly prevalence (<3SD below the mean) is very high at 1% compared to other published estimates. Follow-up of these babies into childhood to measure their developmental outcomes, as well as brain imaging, is therefore important, as recommended by the authors. 

 It would be helpful if the authors could add some further details to the Methods and Results. Can the authors confirm that none of the microcephaly cases had other major malformations or syndromes? Is it possible that there was selective referral of high risk mothers to KCH? What evidence is there that congenital rubella or CMV might be causal factors in this population?

 The study could not confirm circulating Zika in this area of Kenya. In the absence of Zika, it could not be a causal factor for microcephaly. The authors point out that ZIKV was first discovered in East Africa, and the *Aedes Aegypti* mosquito vector is present in Africa. However, the African strain of ZIKV shows different potential for pathogenesis in pregnancy from the Asian/American strain (WHO 2019) ^1^, and the African mosquito also has a different transmission potential (Aubry *et al.* 2018) ^2^, so it should not be assumed that the characteristics of ZIKV as experienced in Latin America would be found in Africa.

I confirm that I have read this submission and believe that I have an appropriate level of expertise to confirm that it is of an acceptable scientific standard.

## References

[1] : Zika Epidemiology Update.2019; Reference source

[2] : Worldwide survey reveals lower susceptibility of AfricanAedes aegypti mosquitoes to diverse strains of Zika virus. *bioRxiv*.2018; 10.1101/342741 10.1101/342741

# Peer review: Reviewer response for version 1

**DOI:** 10.21956/wellcomeopenres.17049.r37999

**Published:** 2020-02-24

**Authors:** Jan Felix Drexler, Thomas Jaenisch

**Affiliations:** 1 Institute of Virology, Charité-Universitätsmedizin Berlin, corporate Member of Freie Universität Berlin, Humboldt-Universität zu Berlin, and Berlin Institute of Health, Berlin, Germany; 2 Colorado School of Public Health, Aurora, CO, USA

*Jan Felix Drexler*

 In the manuscript "Congenital microcephaly unrelated to flavivirus exposure in coastal Kenya” Barsosio and colleagues show their results of an extensive study on the potential role of congenital Zika virus (ZIKV) infections as a cause for microcephaly in Kenya. Although the number of microcephaly cases was high compared to other studies, ZIKV infections were not associated. The study provides very important information about the current role of ZIKV as a teratogenic agent in Africa, where the virus is widespread but reports about congenital malformations due to maternal ZIKV infections are scarce or absent. The size of the sample and the study design are a very good basis for well-founded scientific findings. There are some limitations regarding testing that should be addressed. In general, while Zika infection is not supported by the authors’ results, it cannot be ruled out due to the diagnostic limitations inherent to endemic settings. This should be reflected in the text throughout.

1. ZIKV-specific IgM detection was done by an in-house IgM ELISA assay against the ZIKV NS1 antigen of the prototype strain MR766. It is hard to judge the diagnostic performance of this in-house assay in this particular population and thus the robustness of the authors’ main laboratory findings. Those data must be provided in comparison to other assays. Notably, IgM detection for ZIKV diagnostics suffers from low sensitivity, particularly when targeting NS1-specific antibodies ^1^^-^^3^. Combining detection of IgM/IgA antibodies and of antibodies targeting the ZIKV envelope protein would likely increase sensitivity and thus strengthen the findings of the study.
2. The authors describe how ZIKV FRNT90 assays were conducted. The authors should add a technical description on how FRNT90 was done for dengue virus. In the same line of thought, choice of cell lines and virus stocks differing in the ratio of infectious to non-infectious particles (that still bind antibodies) can dramatically affect NT titers. Comparison of titers is thus always challenging in an endemic scenario in which people get infected multiple times with different flaviviruses. Because conserved epitopes among those multiple flaviviruses cross-stimulate anti-flaviviral immune responses and because different flaviviruses may elicit distinct antibody titer magnitudes, comparison of Zika and dengue NTs in this study deserves to be taken with caution. I agree that since dengue titers were consistently above Zika titers, the NTs are not supportive of Zika infection. I would be cautious though to state that they rule out the latter. In an endemic setting, a higher titer against dengue viruses, likely infecting a person multiple times due to the different co-circulating serotypes, does not rule out a Zika infection eliciting a relatively weaker titer. Solutions in patient diagnostics include testing of consecutive sera from acute and convalescence phases of infection, which is not feasible in this study. Can you compare titers in younger (likely less cumulative flavivirus infections) and older participants?
3. Only cord blood was tested. The authors should perform serologic testing of the mothers for ZIKV-specific IgG. Their-case control design should allow detecting potential ZIKV-specific differences in overall maternal exposure and height of ZIKV-specific neutralizing antibody titers as indirect evidence of potential congenital infection ^4^^,^^5^.
4. As discussed by the authors, the rate of microcephaly was exceptionally high in this study. If not ZIKV, then what is the cause? Both molecular and serologic tests for other pathogens commonly linked to congenital malformations are readily available, including toxoplasmosis, syphilis, rubella virus or herpesviruses ^6^. Confirmation of another aetiology would strengthen the apparent lack of ZIKV.

*Thomas Jaenisch*

 The manuscript provides useful and much needed data about the frequency of microcephaly in Africa, specifically in coastal Kenya, in the absence of ZIKV transmission. It would be great if the authors could provide even more detailed information about the distribution of head circumference in the cohorts they have followed.

 The manuscript should be edited before indexing:

**Title:**

 The potential link to ZIKV infections or flavivirus exposure in the title is misleading, which should be clarified. The authors report that very likely there was no ZIKV transmission during the observation period. The current title leaves room for interpretation in the direction that the microcephaly observed in coastal Kenya is not associated to ZIKV infections, which were present in the region.

 The second weakness of the title is that the authors do not mention ZIKV infection directly in the title (even if ZIKV is the only flavivirus known to cause congenital abnormalities or microcephaly) because the serological results presented to not allow to make this statement. 

**Abstract – conclusions:**

 “Microcephaly was prevalent in coastal Kenya, but does not appear to be related to ZIKV exposure…”

 Same problem as above with the title. The authors continue in the next sentence with “… the ZIKV response observed in our study population was largely due to cross-reactive responses to DENV or other related flaviviruses”. However, this still leaves room for interpretation. The most likely scenario here is that there was simply no ZIKV transmission during the observation period.

**Methods – statistical analysis**

 5 ^th^ line of statistical analysis: there seems to be a word missing “… using INTERGROWTH-21 ^st^ (I21) newborn size-reference charts ….., and standards for birth…”. Which standards?

 The authors chose to compare microcephaly < 3SD (cases) with -2SD to +2SD (controls). Why did they not choose -1SD to +1SD, which would still have resulted in a big enough N for the controls? The reason for this question is that it currently is not clear how many ‘controls’ are close to -2SD (just above the cut-off) and could actually be counted as microcephaly or at least borderline according to some definitions. 

 This reviewer thinks that this warrants a sensitivity analysis with the definition of -1SD - +1SD for controls.

**Results and Figures**

 In Figure 1, all study participants should be accounted for. However, we lack the number of the babies born with the head circumference between -3SD and -2SD (and potentially plus 3SD). Please include this in a footnote to the figure.

 Figure 2 is important to understand the distribution of head circumference in the cohorts, and within different sub-populations. Please include the ‘-1 to +1’ category. In addition, it would be interesting to include a histogram with the actual distribution of head circumference according to SD to really see the spread, and if the distribution approximates a normal distribution or not.

**Discussion**

 The authors stratified the results according to the ascertainment of gestational age – ‘scanned’ versus ‘last menstrual period’. It is interesting to see the effect size of the potential biases resulting from the different methods and this reviewer would encourage the authors to extend the discussion around this point.

We confirm that we have read this submission and believe that we have an appropriate level of expertise to confirm that it is of an acceptable scientific standard, however we have significant reservations, as outlined above.

## References

[1] : Differential Shedding and Antibody Kinetics of Zika and Chikungunya Viruses, Brazil. *Emerg Infect Dis*.25(2) : 10.3201/eid2502.180166 311-315 10.3201/eid2502.180166 30666934PMC6346451

[2] : Added value of IgA antibodies against Zika virus non-structural protein 1 in the diagnosis of acute Zika virus infections. *J Virol Methods*.267: 10.1016/j.jviromet.2019.02.005 8-15 10.1016/j.jviromet.2019.02.005 30779938

[3] : Diagnostic performance of commercial IgM and IgG enzyme-linked immunoassays (ELISAs) for diagnosis of Zika virus infection. *Virology Journal*.2018;15(1) : 10.1186/s12985-018-1015-6 10.1186/s12985-018-1015-6 30005683PMC6045861

[4] : Evidence for Congenital Zika Virus Infection From Neutralizing Antibody Titers in Maternal Sera, Northeastern Brazil. *The Journal of Infectious Diseases*.2017;216(12) : 10.1093/infdis/jix539 1501-1504 10.1093/infdis/jix539 PMC585337329272526

[5] : Risk of Zika microcephaly correlates with features of maternal antibodies. *J Exp Med*.2019;216(10) : 10.1084/jem.20191061 2302-2315 10.1084/jem.20191061 31413072PMC6781003

[6] : TORCH Infections. Toxoplasmosis, Other (syphilis, varicella-zoster, parvovirus B19), Rubella, Cytomegalovirus (CMV), and Herpes infections. *Curr Womens Health Rep*.2002;2(4) :253-8 12150751

# Peer review: Reviewer response for version 1

**DOI:** 10.21956/wellcomeopenres.17049.r37617

**Published:** 2020-02-07

**Authors:** Lavinia Schuler-Faccini

**Affiliations:** 1 Postgraduate Program in Genetics and Molecular Biology, Federal University of Rio Grande do Sul (UFRGS), Porto Alegre, Brazil

This is a manuscript analyzing the prevalence of microcephaly at birth in Coastal Kenya and whether ZIKV could have a role in the etiology of the cases of microcephaly. The authors used data from 11,061 newborns from a County Hospital from 2012 to 2016. They observed a high prevalence of 1% of microcephaly (defined as HC Z-score <3 SD from the mean for GA and sex) in scanned pregnancies. ZIKV was not identified as one of the causes and the only covariate significantly associated was SGA. The authors discuss the limitations of their work but the high prevalence of severe microcephaly is most probably real. Therefore this is an important manuscript to be indexed, and I agree with the authors in their conclusion that further research into potential causes and the clinical consequences of microcephaly in this population is urgently needed.

I confirm that I have read this submission and believe that I have an appropriate level of expertise to confirm that it is of an acceptable scientific standard.
